# Exopolysaccharides isolated from *Rhizopus nigricans* induced colon cancer cell apoptosis *in vitro* and *in vivo* via activating the AMPK pathway

**DOI:** 10.1042/BSR20192774

**Published:** 2020-01-14

**Authors:** Yan Lu, Xiujuan Zhang, Jiayue Wang, Kaoshan Chen

**Affiliations:** 1 School of Life Science, Shandong University, Qingdao 266000, China; 2 National Glycoengineering Research Center, Shandong University, Qingdao 266000, China; 3 Anhui Provincial Engineering Research Center for Polysaccharide Drugs, Anhui Province Key Laboratory of Active Biological Macromolecules, Drug Research and Development Center, School of Pharmacy, Wannan Medical College, Wuhu 241002, China

**Keywords:** AMPK, Colorectal cancer, Exopolysaccharide, JNK-p53, mTORC1, Rhizopus nigricans

## Abstract

Colorectal cancer (CRC) is a leading cause of cancer-related human deaths. The exopolysaccharide (EPS1-1), isolated from *Rhizopus nigricans*, has been described as exhibiting anti-tumor and pro-apoptotic activity against CRC, although the underlying mechanism is poorly understood. Herein, we investigate how EPS1-1 induces apoptosis of CRC cells *in vitro* and *in vivo*. Our results show that, *in vitro*, EPS1-1 suppressed cell growth and facilitated apoptosis in a dose- and time-dependent manner by activating the AMP-activated protein kinase (AMPK) pathway in mouse colon cancer CT26 cells. However, treatment with small interfering RNAs (siRNAs) targeting AMPKα or with compound C, an AMPK inhibitor, interfered with the pro-apoptosis effects of EPS1-1. We also show that EPS1-1 initiated the release of reactive oxygen species (ROS) and liver kinase B1 (LKB1), both of which are necessary signals for AMPK activation. Furthermore, EPS1-1-mediated apoptosis is regulated by inactivation of mammalian target of rapamycin complex 1 (mTORC1) and activation of the jun-NH_2_ kinase (JNK)-p53 signaling axis dependent on AMPK activation. *In vivo*, azoxymethane/dextran sulfate sodium (AOM/DSS)-treated CRC mice, when administered EPS1-1, exhibited activation of the AMPK pathway, inhibition of mTORC1, and accumulation of p53 in tumor tissues. Collectively, these findings suggest that EPS1-1-induced apoptosis relies on the activation of the AMPK pathway. The present study provides evidence suggesting that EPS1-1 may be an effective target for development of novel CRC therapeutic agents.

## Introduction

Colorectal cancer (CRC) is one of the most common malignancies and leading causes of cancer-related morbidity and mortality worldwide [1]. Although current conventional chemotherapeutic methods for CRC treatment can prolong life expectancy, they also elicit multiple adverse side effects that significantly impact patients’ quality of life, and the prognosis of patients with this malignancy has not improved [2]. Therefore, there is an urgent need to explore novel substances as therapeutic agents against CRC with few or no adverse side effects. Fortunately, several studies have shown that naturally active polysaccharides, extracted from fungi, plants, animals, algae, and lichens [3–6], possess anti-CRC properties [7,8] through a variety of mechanisms.

AMP-activated protein kinase (AMPK), a conserved serine/threonine kinase, is nearly ubiquitous in eukaryotes and is regulator of cellular energy and nutrient uptake in response to cellular stress, including hypoxia, exercise, and starvation [9]. AMPK is a heterotrimeric complex, and is composed of a catalytic α subunit and regulatory β and γ subunits. The activity of AMPK is regulated by the AMP/ATP ratio as well as by the phosphorylation of the α subunit at Thr^172^ [10]. A number of studies have shown that AMPK effectively regulates mitochondrial biogenesis and disposal [11], autophagy [12], and cellular polarity [13]. Liver kinase B1 (LKB1) is one of the upstream kinases for AMPK, and the ability of LKB1 to bind and phosphorylate AMPK is affected by the phosphorylation of LKB1 [14]. Phosphorylation of acetyl-CoA carboxylase (ACC) is a reliable indicator for the activation of AMPK and is an important downstream signal of AMPK [15]. Importantly, AMPK also plays a vital role in the induction of apoptosis in various types of cancer cells [16]. Liu et al. [17] reported that SEC-induced activation of Annexin (ANXA7) GTPase inhibits prostate cancer metastasis through AMPK activation. Moreover, a recent paper revealed that berberine regulates AMPK signaling pathways and suppresses colon tumorigenesis in azoxymethane/dextran sulfate sodium (AOM/DSS)-induced CRC mice [18]. Additional studies have reported that diindolylmethane, and its halogenated derivatives, promote protective autophagy in human prostate cancer cells via induction of the oncogenic protein, astrocyte elevated gene-1 (AEG-1) and activation of AMPK [19]. These findings suggest that regulation of the AMPK pathway is a potential therapeutic target for cancer. Nevertheless, to date, very little attention has been given to examine the effect that natural active polysaccharides have on the AMPK pathway.

*Rhizopus nigricans*, a zygomycete filamentous fungus, is widely used in the pharmaceutical industry [20]. In our previous studies, extracellular polysaccharide (EPS1-1), isolated from the fermentation broth of *R. nigricans*, had an average molecular mass of 9682 Da, and was found to be composed of glucose, mannose, galactose, and fructose at a molar ratio of 5.89:3.64:3.20:1.00, respectively [21]. Moreover, EPS1-1 was found to potentially improve immunity [22], and inhibit the growth of xenograft tumors via regulation of the mitochondrial pathway [21,23,24]. EPS1-1 also suppressed the occurrence and development of AOM/DSS-induced CRC in mice. However, we did not further elucidate the anticancer molecular mechanisms employed by EPS1-1 [25]. Thus, in the present study, we sought to reveal the potential anti-CRC mechanisms of EPS1-1 both in *in vitro* and *in vivo* models.

## Materials and methods

### Chemicals and reagents

The reactive oxygen species (ROS) assay kit was provided by Beyotime Institute of Biotechnology (Shanghai, China). The 5× SDS protein sample buffer (WB-0091) was purchased from Dingguo Corp (Beijing, China). 5-Aminoimidazole-4-carboxamide-1-β-d-ribofuranoside (AICAR), Compound C, and SP600125 were obtained from APExBIO (U.S.A.). Rapamycin was procured from Solabio (China).

*R. nigricans* was isolated from straw and preserved in the Laboratory of Biomass Resources at the Shandong University (Qingdao, China). EPS1-1 was obtained according to the previously reported method [21].

### Cell culture

CT26 cells were obtained from Wannan Medical College (Wuhu, China) and cultured in Roswell Park Memorial Institute (RPMI)-1640 media (Gibco GRL, U.S.A.), supplemented with 10% fetal bovine serum (FBS; Gibco GRL, U.S.A.) and 1% penicillin/streptomycin (Gibco GRL, U.S.A.) in a humidified atmosphere of 5% CO_2_ at 37°C.

### Cell viability assay

The cytotoxicity of EPS1-1 was measured using a 3-(4,5-dimethylthiazol-2-yl)-2,5-diphenyltetrazolium bromide (MTT) assay [26]. Briefly, CT26 cells were seeded in 96-well plates and treated with various concentrations of EPS1-1 for 36 h; 20 μl MTT (5 mg/ml) was then added to each well, and the samples were incubated for 4 h at 37°C. The supernatants were removed carefully, and 150 μl of dimethyl sulfoxide (DMSO) was used to solubilize the formazan. Optical densities were measured using an automatic microplate reader at 570 nm. The cell viability was calculated as the percentage of viable cells in the treated group compared with the non-treated group.

### ROS measurement

ROS levels were determined with 2′,7′-dichlorofluorescein diacetate (DCFH-DA) as previously described [27]. Briefly, following treatment with EPS1-1, CT26 cells were incubated with 10 mM of DCFH-DA at 37°C for 20 min in the dark and washed three times with phosphate buffered saline (PBS). Stained cells were then visualized using a fluorospectro-photometer at an excitation wavelength of 488 nm and an emission wavelength of 535 nm.

### Quantification of apoptosis by ELISA

The Cell Apoptosis ELISA Detection Kit (Roche, Palo Alto, CA) was used according to manufacturer’s instructions to analyze the rate of apoptosis in CRC cells following treatment with EPS1-1. Briefly, after the indicated treatments were applied, the cytoplasmic histone/DNA fragments were extracted from cells and bound to immobilized anti-histone antibody. Subsequently, the peroxidase–conjugated anti-DNA antibody was used for the detection of immobilized histone/DNA antibody fragments. After the addition of the peroxidase substrate, spectrophotometric absorbances of the samples were determined using Epoch 2 Microplate reader at 405 nm.

### Western blotting

The concentration of extracted protein was measured using a BCA Protein Assay Kit (Beyotime, Nanjing, China). Equal amounts of protein were separated by 10% sodium dodecyl sulfate/polyacrylamide gel electrophoresis (SDS/PAGE) and subjected to Western blotting analysis using specific primary antibodies (Supplementary Table S1). Finally, antibody binding was detected using an enhanced chemiluminescence (ECL; Thermo Fisher Scientific) detection system in the dark. Positive immunoreactive bands were quantified by densitometric analysis using ImageJ software (NIH, Bethesda, MD, U.S.A.) and compared with those of the control.

### Transient transfection of small interfering RNAs

Small interfering RNAs (siRNA) were synthesized by Gene Pharma (Shanghai, China) and are presented in Table 1. Cells (3 × 10^5^) were seeded in a six-well plate with antibiotic-free RPMI media and incubated for 6 h. The targeting siRNAs were transfected using Lipofectamine2000 Transfection Reagent (Dingguo Corp., Beijing, China; GL3413-50UL) according to manufacturer’s instructions. After incubation for an additional 6 h, the cells were treated with EPS1-1 for 36 h and analyzed by Western blot analysis.

**Table 1:** siRNAs sequences

siRNA	Sense	Antisense
AMPKα	5′-GGGAACACGAGUGGUUUAATT-3′	5′-GGAGAGCUAUUUGAUUAUATT-3′
LKB1	5′-GAGGGAUGUUGGAGUAUGATT-3′	5′-UCAUACUCCAACAUCCCUCTT-3′
mTOR	5′-CCGGCACACAUUUGAAGAATT-3′	5′-UUCUUCAAAUGUGUGCCGGTT-3′

### Colon tissues obtained from AOM/DSS-induced CRC

Previous studies have reported that EPS1-1 inhibits tumor growth in AOM/DSS-induced CRC [25]. Our animal study was performed in accordance with the regulations for the administration of Affairs Concerning Experimental Animals of China and the Ethics Committee of Shandong University with the approval number SYDWLL-2018-12 (Qingdao, China). Thirty SPF male BALB/c mice (4–6 weeks old, average body weight 20 g) were purchased from Beijing Vital River Laboratories Co. (Beijing, China). All mice were fed in the SPF animal house of Shandong University (Qingdao, China). Briefly, the mice were randomized into three groups: Control group (*n*=10), free access to regular water and food; Model group (*n*=10), intraperitoneally injected with AOM (10 mg/kg) and, after a week, fed 2.5% DSS for a week and normal water for 2 weeks with four cycles; EPS1-1 group (*n*=10), based on the Model group, orally treated with EPS1-1 (180 mg/kg) daily from the first day of the first cycle to the end. Finally, mice were killed by the method of cervical dislocation and intestinal tissue were collected and stored at −80°C for the subsequent experiments.

### Histopathological analysis of mice

The colon of each mouse was washed with PBS buffer to remove intestinal contents and photographed. Next, the colon was opened longitudinally, placed flat on a plate, and observed using a stereo microscope.

Colon tissue was transversely cut into 5-mm sections, fixed in 10% formaldehyde overnight and then stored in 70% ethanol. Embedded colonic tissues in paraffin were cut into 5-μm slices and placed flat on slides. Next, slides were stained with Hematoxylin/Eosin (H&E, Beyotime, Shanghai, China) for microscopic observation and histological analysis.

### Protein extraction of mouse colon tissue in mice

Colon tissue samples (40 mg) were homogenized in radioimmunoprecipitation (RIPA) lysis buffer containing phosphatase protease inhibitor cocktail (Roche Diagnostics, Indianapolis, IN) and protease inhibitor (PMSF) and lysed for 30 min on ice. The lysates were then centrifuged at 12000×***g*** for 40 min, and the supernatants, which contained the protein fraction, were collected in a new 1.5 ml centrifuge tube. Protein concentration was measured using a BCA Protein Assay Kit. Next, proteins in colon tissues from the Control, Model, and EPS1-1 groups were analyzed using Western blotting.

### Statistical analysis

All experimental data in the present study were performed in triplicate. The significance of differences was determined by one-way analysis of variance (ANOVA) with a post-hoc analysis (>two groups) or by Student’s *t* tests (two groups). *P*-values <0.05 were considered statistically significant.

## Results

### EPS1-1 inhibits CT26 cell proliferation and induces apoptosis

**Figure 1:**
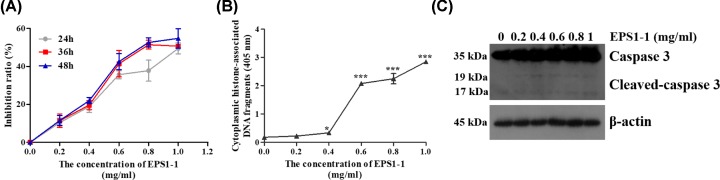
The effect of EPS1-1 on cell viability and apoptosis in CT26 cells Cells were exposed to different concentrations of EPS1-1 (0, 0.2, 0.4, 0.6, 0.8, and 1 mg/ml) for 24, 36 and 48 h. (**A**) Cell viability of CT26 cells was determined using MTT assays. (**B**) Histone-DNA ELISA assays were performed to examine the rate of apoptosis in CT26 cells. (**C**) Western blotting was used to detect the expression of caspase 3 protein. Experiments were repeated at least five times. *, *P*<0.05, ***, *P*<0.005 versus untreated group (ANOVA).

In order to assess the effect of EPS1-1 on cell proliferation, CT26 cells were exposed to different concentrations of EPS1-1 (0, 0.2, 0.4, 0.6, and 1 mg/ml) for 24, 36 and 48 h, followed by MTT assay. As illustrated in Figure 1A, EPS1-1 significantly increased the inhibition rate of CT26 cells at different concentrations in a time-dependent manner. In addition, Histone-DNA ELISA assays were used to evaluate apoptosis in CT26 cells. After treatment with EPS1-1 for 36 h, we found that EPS1-1 induced apoptosis of CT26 cells in a dose-dependent manner (Figure 1B). Moreover, the expression of caspase 3 protein was significantly increased and cleaved by the treatment with EPS1-1 (Figure 1C). These data suggest that EPS1-1 could increase the inhibition rate and induce apoptosis in CT26 cells.

### Effect of EPS1-1 on AMPK pathway in CT26 cells

**Figure 2:**
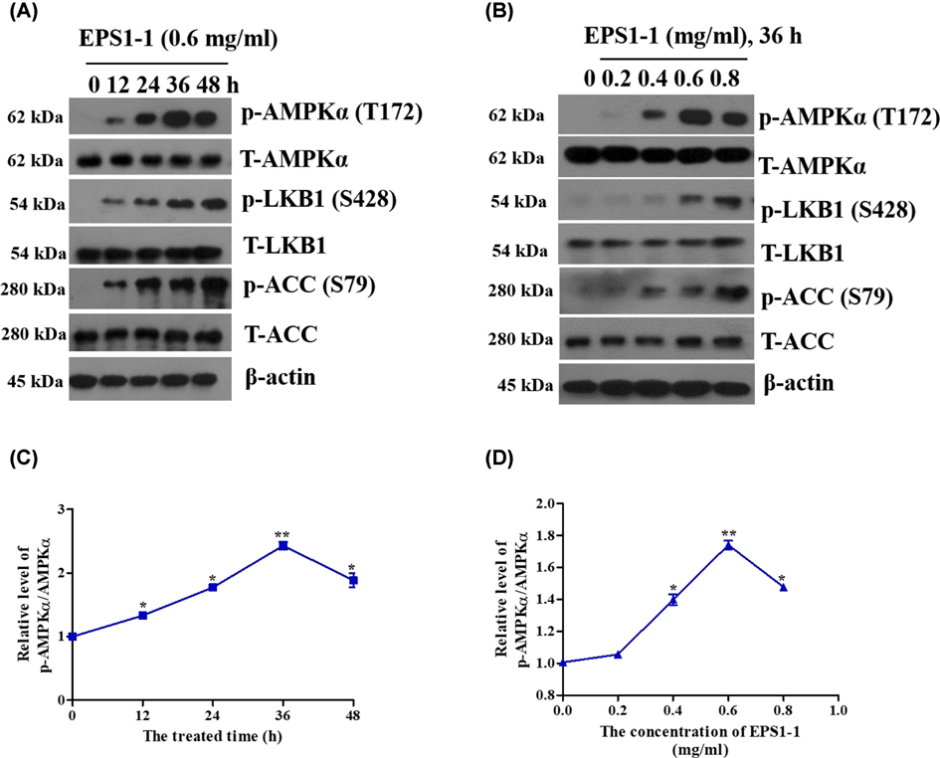
EPS1-1 induces AMPK activation in CT26 cells (**A,B**) CT26 cells were treated with EPS1-1 (0.6 mg/ml) and cultured for 0, 12, 24, 36, and 48 h; or treated with different doses of EPS1-1 (0, 0.2, 0.4, 0.6, and 0.8 mg/ml) and cultured for 36 h. T-AMPKα, p-AMPKα (Thr^172^), T-LKB1, p-LKB1 (S428), T-ACC, p-ACC (S79), and β-actin were detected via Western blot analysis. (**C,D**) AMPKα phosphorylation was quantified using ImageJ. Experiments were repeated at least three times. *, *P*<0.05, **, *P*<0.01, versus untreated group (ANOVA).

AMPK serves as a potent target for anti-tumor treatment. We, therefore, sought to investigate the effects of EPS1-1 on AMPK. Western blotting analysis revealed that the phosphorylation of AMPKα, LKB1 (AMPK upstream effector), and ACC (AMPK downstream molecule), was increased following treatment with EPS1-1 compared with the control cells in both time- and dose-dependent manner (Figure 2A,C and B,D). However, the total protein level of AMPKα, LKB1, and ACC was not changed. The increased phosphorylation of LKB1, AMPKα, and ACC was observed at 12 h after EPS1-1 treatment and continued for 48 h. ACC is a target of AMPK, and has been described as a reliable indicator of AMPK activation. Therefore, these observations indicate that the AMPK pathway was activated by EPS1-1 treatment.

### EPS1-1 reduced proliferation and induced apoptosis of CT26 cells via AMPK pathway

**Figure 3:**
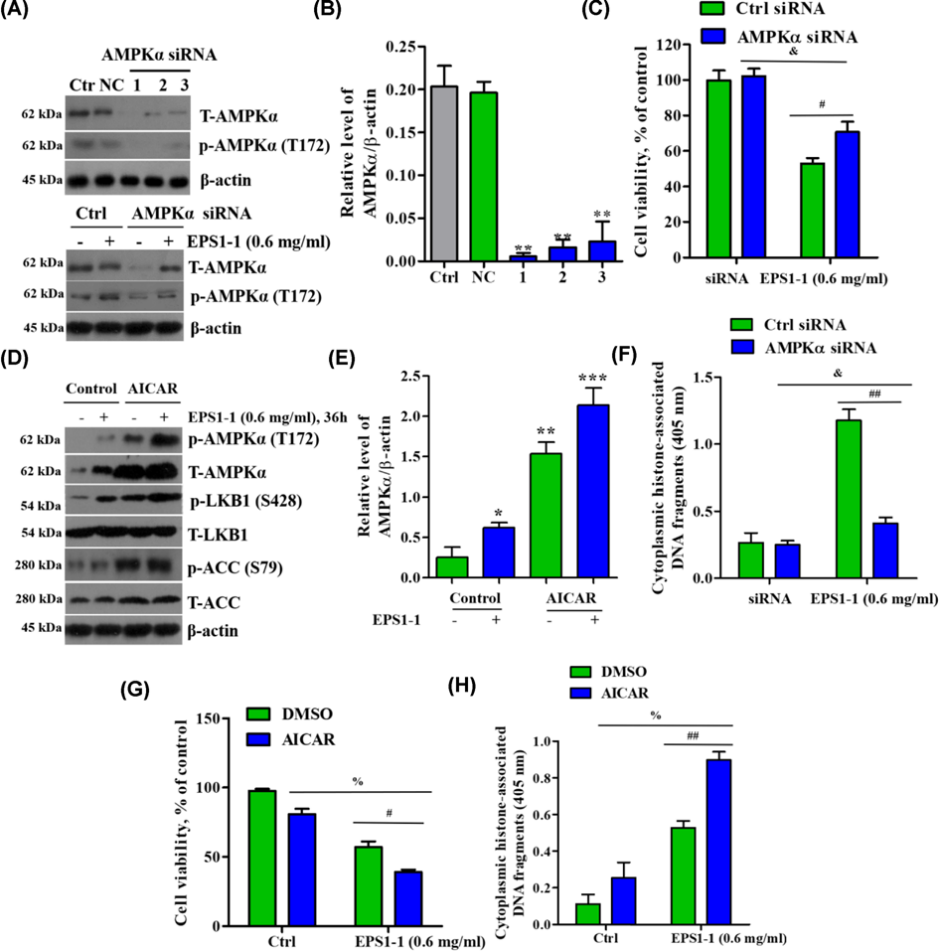
AMPK activation is required for EPS1-1-induced cell death *in vitro* (**A**) CT26 cells were transfected with control (scrambled) or AMPKα siRNA for 48 h. AMPKα, p-AMPKα, and β-actin expression levels were detected by Western blot analysis. (**B,E**) AMPKα phosphorylation was quantified using ImageJ. (**C**) Cells with AMPKα knocked down were treated with 0.6 mg/ml EPS1-1 for 36 h. Cell viability was detected using MTT assay. (**D**) CT26 cells were pretreated with an AMPK activator, AICAR (1 mM) followed by 0.6 mg/ml EPS1-1. (**F**) Cell apoptosis was detected by Histone/DNA ELISA. (**G**) Cell viability was detected by MTT assay after 36 h. (**H**) Cell apoptosis was detected by histone/DNA ELISA after 36 h. Experiments were repeated at least three times. *, *P*<0.05, **, *P*<0.01, ***, *P*<0.005 versus untreated group; ^#^, *P*<0.05, ^##^, *P*<0.01 versus EPS1-1-treated group in control group; ^&^, *P*<0.01 versus EPS1-1-treated group in AMPKα siRNA group; ^%^, *P*<0.01 versus EPS1-1-treated group in AICAR group (ANOVA).

The above results exhibited that EPS1-1 activated the AMPK pathway in CT26 cells. We, therefore, sought to further verify and define the contribution of AMPK in EPS1-1-induced apoptosis and reduced proliferation in CT26 cells. The expression of AMPKα in CT26 cells was found to be significantly decreased by >90% after transient transfection with AMPKα siRNA, and the phosphorylation of AMPKα was also suppressed (Figure 3A,B); whereas EPS1-1 treatment increased AMPKα expression and AMPKα phosphorylation (Figure 3A). We then used an activator of AMPK signaling, AICAR, to overexpress AMPKα, and showed that AMPKα expression and AMPK phosphorylation were dramatically increased in CT26 cells (Figure 3D,E). Subsequently, we evaluated cell proliferation and apoptosis using MTT assays and Histone/DNA ELISA, respectively. MTT analysis revealed that EPS1-1 significantly reduced the level of cell proliferation (reduced cellular viability) that had previously been enhanced following silencing of AMPKα expression (Figure 3C). Moreover, EPS1-1 further amplified the effect that overexpression of AMPKα had in a significant decrease in cell proliferation (Figure 3G). Histone/DNA ELISA results showed that EPS1-1 effectively induced apoptosis in the presence of AMPKα (Figure 3F,H). Together, these observations suggest that EPS1-1 may effectively promote the inhibitory effect of AMPK on CT26 cells.

### Effect of ROS on EPS1-1-induced AMPK activation

**Figure 4:**
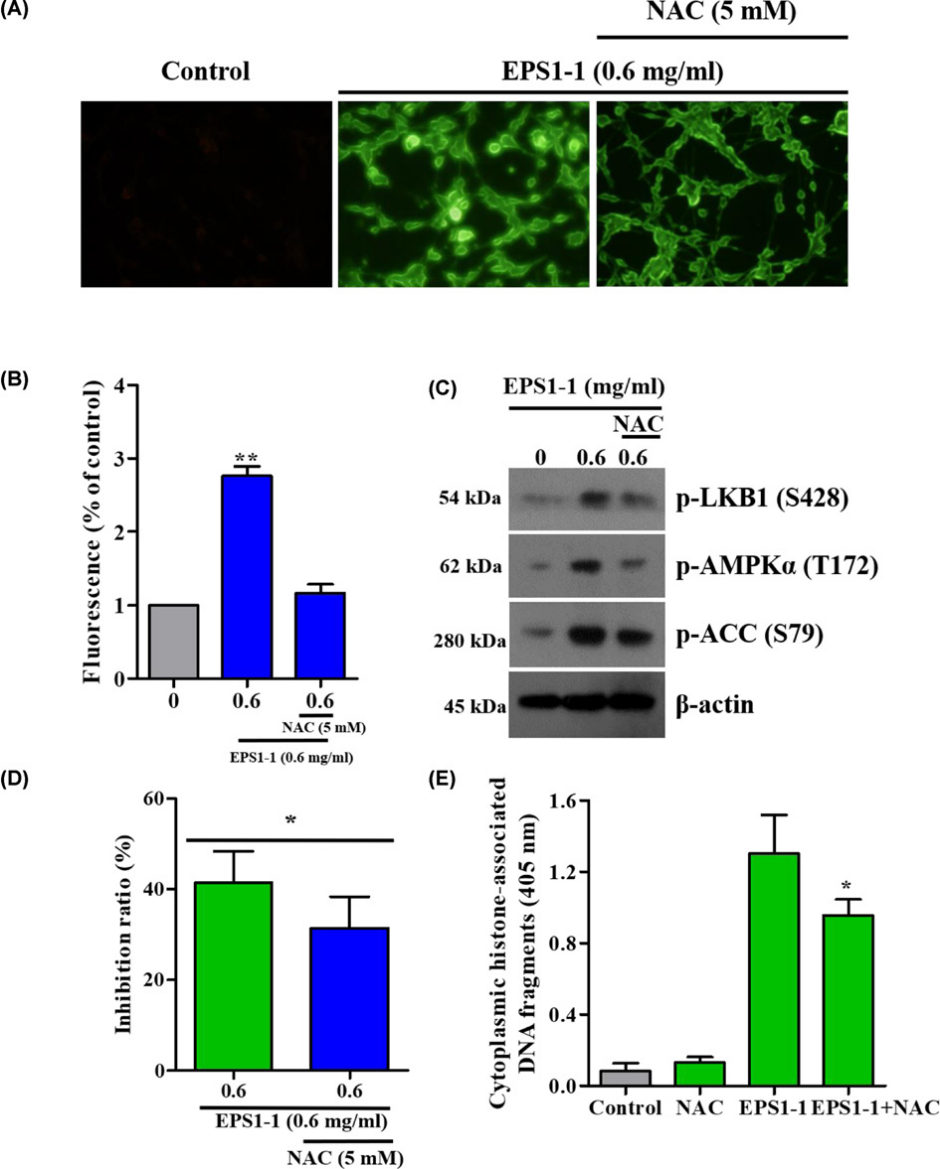
ROS is involved in EPS1-1-induced AMPK activation (**A**) After treatment with 0.6 mg/ml of EPS1-1 in the absence or presence of NAC (5 mM) for 1 h, the ROS levels in CT26 cells were quantified using DCF-DA, a fluorescent dye, and an ultraviolet spectrophotometer. (**B**) ROS level was quantified using ImageJ. (**C**) CT26 cells were treated with the indicated concentration of EPS1-1 in the absence or presence of NAC (5 mM) for 36 h. Phosphorylation levels of AMPKα, LKB1, and ACC were determined by Western blotting. (**D**) Cell viability was detected by MTT assay. (**E**) Cell apoptosis was detected by Histone/DNA ELISA. Experiments were repeated at least three times. *, *P*<0.05, **, *P*<0.01 versus control group.

It is well established that ROS, is a strong activator of AMPK, and has an important role in apoptosis [27,28,29], thus, we wondered whether ROS production is related to EPS1-1-induced AMPK activation. To evaluate the effect of EPS1-1 on ROS activity, we examined the ROS level in CT26 cells by DCF-DA staining. Compared with the control cells, treatment with 0.6 mg/ml of EPS1-1 significantly increased the production of ROS in CT26 cells (Figure 4A,B). Furthermore, Western blot results showed that treatment with N-acetyl-l-cysteine (NAC), an ROS scavenger, significantly inhibited EPS1-1-induced phosphorylation of AMPKα, LKB1, and ACC (Figure 4C), suggesting that ROS was required in the process of EPS1-1-induced AMPK activation. To determine the effect of ROS on EPS1-1-induced apoptosis, we measured the viability and rate of apoptosis in cells treated with NAC in the presence of EPS1-1. We found that the viability (MTT assay) of CT26 increased and the level of apoptosis was significantly decreased following treatment with NAC, whereas EPS1-1 effectively reversed these effects (Figure 4D,E). These findings indicate that ROS participates in EPS1-1-induced activation of AMPK to further regulate CT26 cell apoptosis.

### LKB1 is involved in EPS1-1-induced AMPK activation

**Figure 5:**
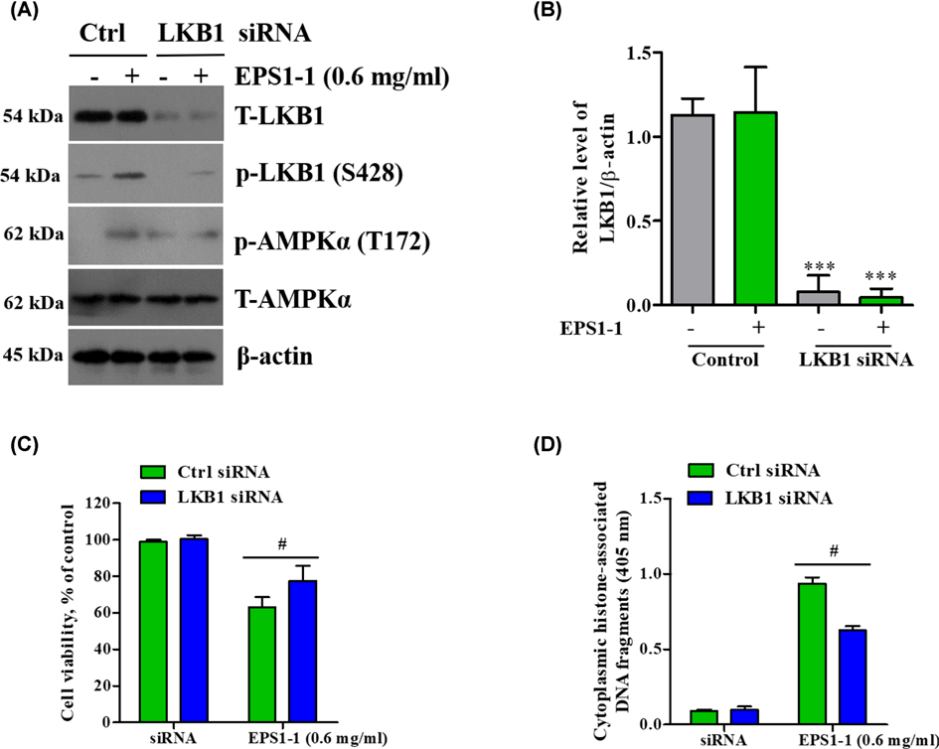
LKB1 is involved in EPS1-1-induced AMPK activation (**A**) CT26 cells were transfected with LKB1 or scramble siRNAs and treated with 0.6 mg/ml of EPS1-1. LKB1 and AMPK activation were determined using Western blotting. (**B**) LKB1 expression was quantified using ImageJ. (**C**) Cell viability was examined by MTT assay and (**D**) cell apoptosis was detected by histone/DNA ELISA. Experiments were repeated at least three times. ***, *P*<0.005 versus untreated group; ^#^, *P*<0.05 versus EPS1-1-treatment group in Ctrl cells (ANOVA).

LKB1, a tumor suppressor, is one of the major upstream kinases involved in AMPK activation [30,31]. Our results have shown that EPS1-1 induces the phosphorylation of LKB1 while activating the AMPK pathway. We, therefore, further explored the relationship between LKB1 and EPS1-1-induced apoptosis and AMPK activation using target-specific RNA interference. As expected, silenced expression of LKB1 significantly suppressed AMPK phosphorylation compared with the control siRNA-transfected cells (Figure 5A,B), whereas EPS1-1 treatment caused a slight increase in the level of AMPKα phosphorylation (Figure 5A). In addition, silencing of LKB1 diminished the previously observed effect induced by EPS1-1 in decreasing cell viability and enhancing apoptosis (Figure 5C,D). These findings indicate that LKB1 acts as an important regulator of EPS1-1-induced AMPK activation and reduced cell viability/enhanced apoptosis in CT26 cells.

### EPS1-1-induced apoptosis and reduced cell viability is related to inhibition of AMPK-mediated mTOR complex 1

**Figure 6:**
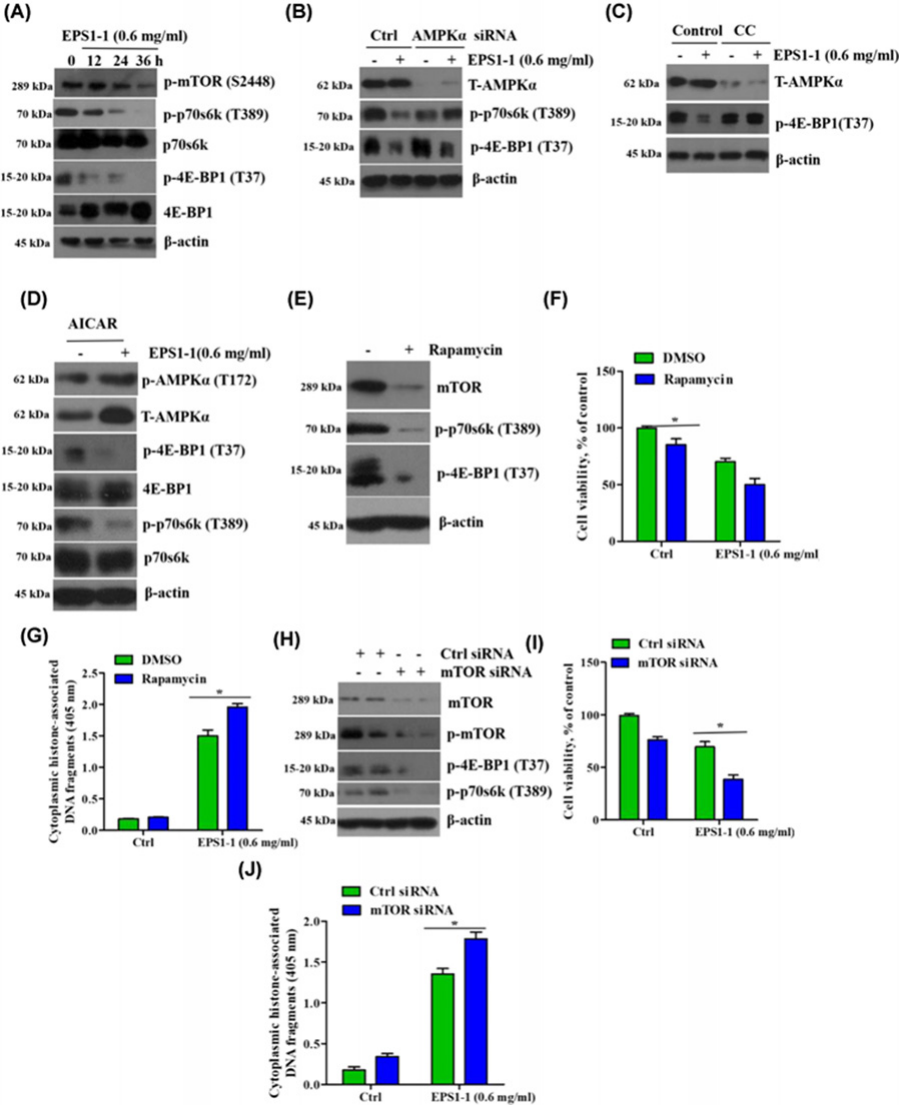
EPS1-1-induced apoptosis and reduced cellular viability is related to AMPK-mediated mTORC1 inhibition (**A**) CT26 cells were treated with 0.6 mg/ml EPS1-1 and cultured for 0, 12, 24, and 36 h. p-mTOR (S2448), p-p70s6k (T389), p70s6k, p-4E-BP1 (T73), and 4E-BP1 were detected via Western blotting. (**B**) CT26 cells with silenced AMPKα were treated with 0.6 mg/ml EPS1-1 for 36 h. T-AMPKα, p-p70s6k (T389), and p-4E-BP1 (T73) were detected. (**C**) After treatment with 5 mM Compound C for 1 h, CT26 cells were treated with 0.6 mg/ml EPS1-1 for 36 h. T-AMPKα and p-p70s6k (T389) were detected. (**D**) Western blotting analysis of mTORC1 activation in CT26 cells following treatment with 1 mM AICAR. (**E** and **H**) CT26 cells were treated with 100 nm rapamycin for 12 h and transfected with mTOR or scramble (control) siRNAs for 48 h. mTOR, p-mTOR (S2448), p-p70s6k (T389), p-4E-BP1 (T73), and β-actin expressions were examined. (**F,I** and **G,J**) Cell viability was examined by MTT assay and apoptosis was detected by histone/DNA ELISA. The experiments were repeated at least three times. *, *P*<0.05, versus the EPS1-1-treated group (ANOVA).

It has been reported that TOR is the central component of a complex signaling network that regulates cell growth and proliferation as well as animal size [32]. Moreover, AMPK has been demonstrated to be a major negative regulator of the mTOR pathway and to regulate tumor cell growth and apoptosis [33–35]. Thus, we next sought to examine whether the inhibitory effect of EPS1-1 on tumor cells was mediated through the AMPK/mTOR signaling pathway. Western blot analysis revealed that silencing of AMPKα expression, mammalian target of rapamycin complex 1 (mTORC1) inhibition induced by EPS1-1 was dramatically reversed, however, this inhibitory effect was enhanced by EPS1-1 treatment (Figure 6A-C). Alternatively, overexpression of AMPKα led to the inactivation of the mTOR pathway (Figure 6D). To further investigate the role of mTORC1 on EPS1-1-induced CT26 cell apoptosis, cells were treated with mTORsiRNA and mTOR inhibitor (Rapamycin) (Figure 6E,H, respectively). The cell viability and apoptosis rates were first measured by MTT analysis and Histone/DNA ELISA, respectively. We found that EPS1-1 treatment caused a significant reduction in viability in mTOR-deficient cells (Figure 6F,I). Furthermore, we determined that the rate of apoptosis markedly increase following treatment with EPS1-1 in mTOR-deficient cells (Figure 6G,J). Taken together, these results indicate that AMPK-mediated mTORC1 inhibition is involved in EPS1-1-induced CT26 cell apoptosis and subsequent reduced viability.

### EPS1-1 activates the JNK-p53 signaling axis though AMPK activation

**Figure 7:**
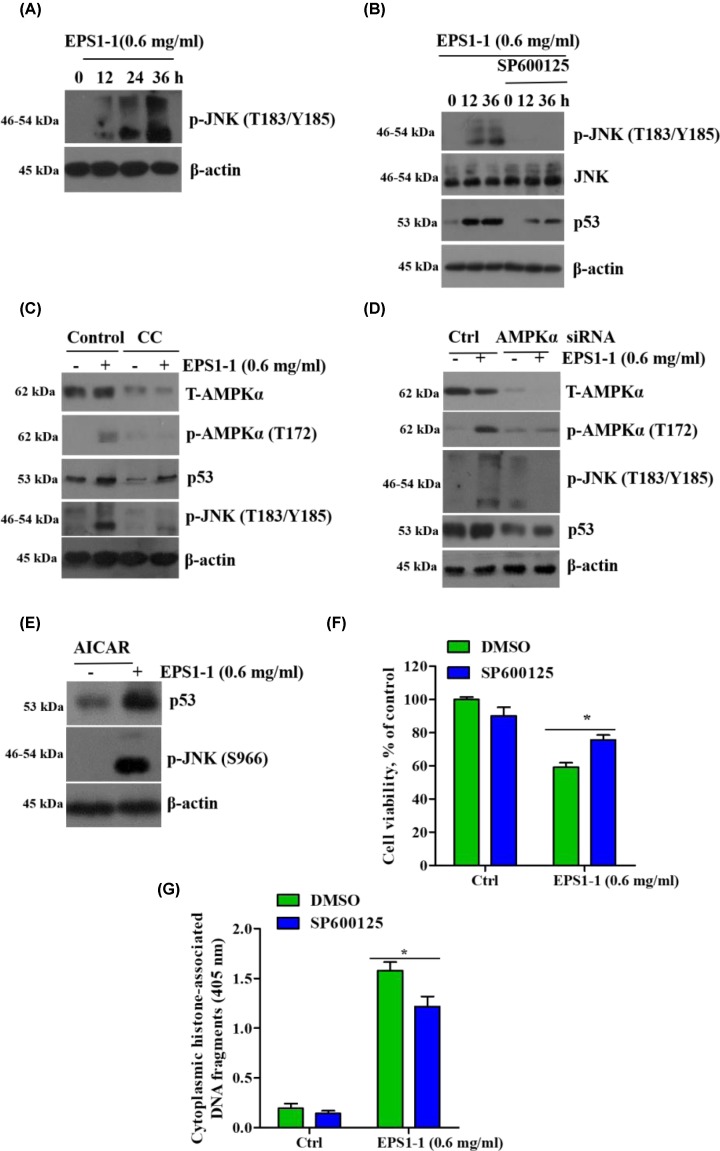
EPS1-1 activates the JNK-p53 signaling axis through AMPK activation (**A**) CT26 cells were treated with 0.6 mg/ml EPS1-1 for the indicated times, and the phosphorylation levels of JNK were examined by Western blotting. (**B**) CT26 cells were treated with 25 μm SP600125 for 1 h followed by treatment with 0.6 mg/ml EPS1-1. The expression of JNK, p-JNK, p53, and β-actin were examined via Western blotting. (**C**) After treatment with 5 mM compound C for 1 h, CT26 cells were treated with 0.6 mg/ml EPS1-1 for 36 h. The expression of T-AMPKα, p-AMPKα, p53, p-JNK, and β-actin were examined via Western blotting. (**D**) CT26 cells transfected with scramble or AMPKα siRNA were treated with 0.6 mg/ml of EPS1-1 and the expression of T-AMPKα, p-AMPKα, p53, p-JNK, and β-actin were examined via Western blotting. (**E**) The results of p53 and p-JNK Western blot analysis following treatment with 1 mM of AICAR. (**F,G**) CT26 cells were treated with 25 μm SP600125 for 1 h followed by 0.6 mg/ml of EPS1-1. Cell viability was examined by MTT assay and apoptosis was detected using histone/DNA ELISA. The experiments were repeated at least three times. *, *P*<0.05, versus control group.

Recent studies have confirmed that activation of the JNK-p53 pathway regulates several important cellular functions including cell growth and apoptosis [36–38]. To determine whether EPS1-1 induces cell apoptosis through p53 and JNK phosphorylation, we examined the effects of EPS1-1 on the JNK-p53 pathway and found that pre-treatment with EPS1-1 significantly up-regulated phosphorylation of JNK and the expression of p53 in CT26 cells (Figure 7A,B). To further assess the relationship between AMPK and the JNK-p53 pathway, we used a specific inhibitor of AMPK, compound C, and AMPKα siRNA to knockdown the expression of AMPKα. We found that both these treatment groups exhibited significantly inhibited-JNK activation and accumulation of p53 (Figure 7C,D). Furthermore, EPS1-1-induced reduction in CT26 cell viability (Figure 7F) and enhancement of apoptosis (Figure 7G) were significantly suppressed by subsequent treatment with the JNK inhibitor, SP600125, indicating that JNK activation may be required for the anticancer activity of EPS1-1. In addition, we found that treatment with the JNK inhibitor, SP600125, significantly reduced the level of EPS1-1-induced p53 accumulation (Figure 7B), whereas this effect was reversed following treatment with AICAR (Figure 7E). These results indicate that EPS1-1-induced AMPK-JNK activation may serve as a key upstream signal for p53 expression. These observations indicate that EPS1-1 induces the activation of AMPK, which subsequently activates the JNK-p53 pathway to mediate CT26 cell viability/apoptosis.

### EPS1-1 inhibits tumor growth in AOM/DSS-induced colon cancer mice

**Figure 8:**
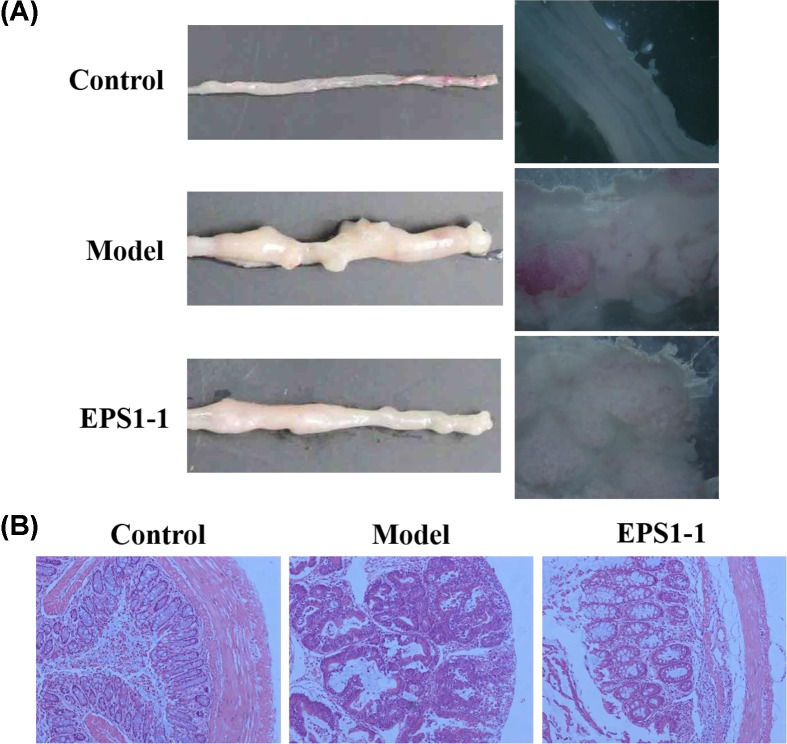
Effects of EPS1-1 on AOM/DSS-induced colon cancer in mice (**A**) Macroscopic structure of the colon in the control, model, and EPS1-1 groups. (**B**) H&E staining of colon tissues observed using an optical microscope.

To demonstrate the anti-tumor activity of EPS1-1, we established a mouse model of colon cancer and the colon structure was observed. As shown in Figure 8A, the number of tumors in the model group was dramatically increased compared with that in the control group, whereas the number of tumors in the EPS1-1 group was lower than that in the model group. In addition, hemorrhagic sites and multiple adenomas were observed in colon tissues from the model group, whereas these adverse symptoms were significantly alleviated in the EPS1-1 group (Figure 8A). Moreover, histopathology results showed that some characteristic pathological symptoms induced by AOM/DSS, such as disruption of epithelial mucous layer structure, gland hyperplasia, large and hyperchromatic nucleus, increased nucleoplasmic ratio, and inflammatory cell infiltration, were significantly reduced by EPS1-1 (Figure 8B). These observations demonstrate that EPS1-1 could decrease tumorigenesis in AOM/DSS-induced colon cancer mice.

### EPS1-1 attenuated tumorigenesis in AOM/DSS-induced CRC through AMPK activation

**Figure 9:**
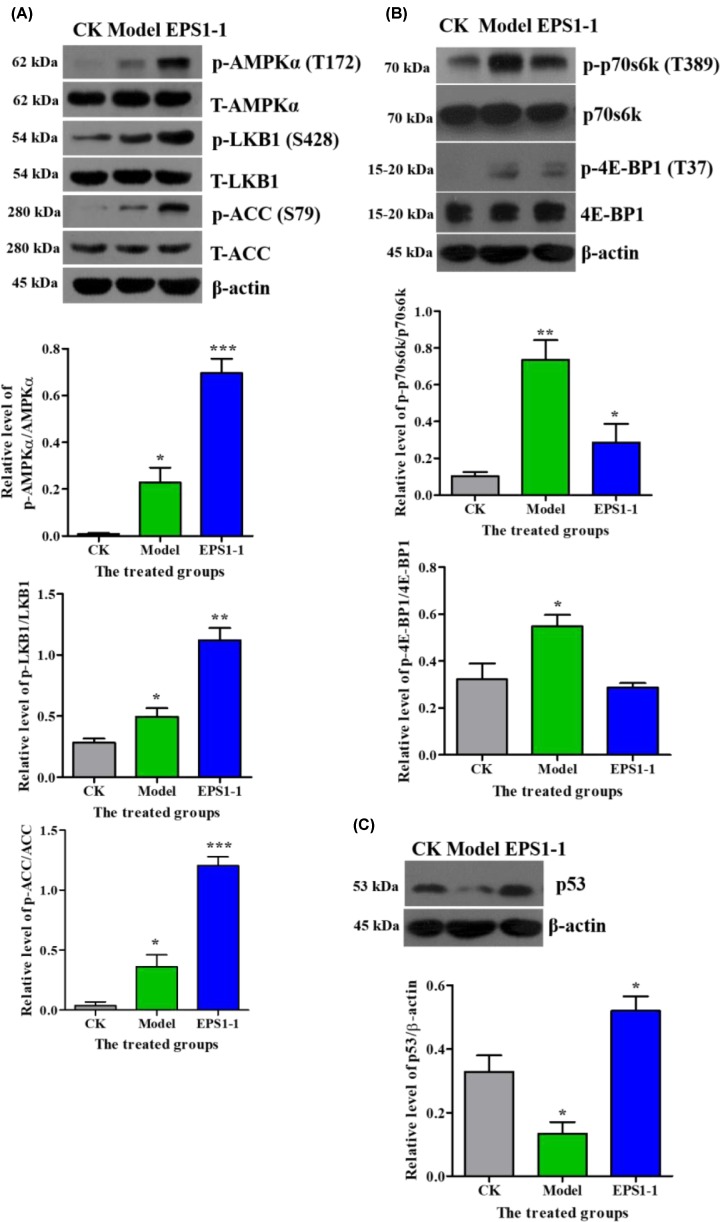
AMPK signaling pathway was involved in the anti-tumor effect elicited by EPS1-1 in AOM/DSS-induced CRC (**A**) Western blotting analysis was used to quantify the expression of AMPKα, LKB1, and ACC as well as that of phosphorylated AMPKα, LKB1, and ACC. (**B**) p70s6k, 4E-BP1, and β-actin expression as well as phosphorylated p70s6k and 4E-BP1 were quantified via Western blotting. (**C**) The expression of the tumor suppressor gene *p53* was quantified via Western blotting. CK: the control group; Model: the model group; EPS1-1: the EPS1-1 group. Data are represented as the mean from three independent experiments. Standard deviations (SD) are less than 10%. *, *P*<0.05, **, *P*<0.01, ***, *P*<0.005 *vs.* CK group.

We demonstrated that EPS1-1 inhibited the proliferation of CT26 cells *in vitro* through AMPK activation. However, previous studies have shown that EPS1-1 significantly inhibited the occurrence and development of AOM/DSS-induced CRC [25]. Thus, to expand on our *in vitro* observations, we further investigated whether EPS1-1 inhibited the growth of tumor through AMPK activation *in vivo*. As shown in Figure 9A, the mice treated with EPS1-1 exhibited significantly increased levels of AMPKα, LKB1, and ACC phosphorylation compared with the disease model mice. Moreover, treatment with EPS1-1 served to consistently suppress the phosphorylation level of p70s6k and 4E-BP1 (Figure 9B). Meanwhile, the tumor suppressor gene *p53* was highly expressed in the EPS1-1 groups (Figure 9C). These *in vivo* results are consistent with those observed *in vitro*. Thus, these findings further confirmed that the anti-tumor effect of EPS1-1 *in vivo* and *in vitro* was associated with activation of the AMPK pathway.

## Discussion

**Figure 10:**
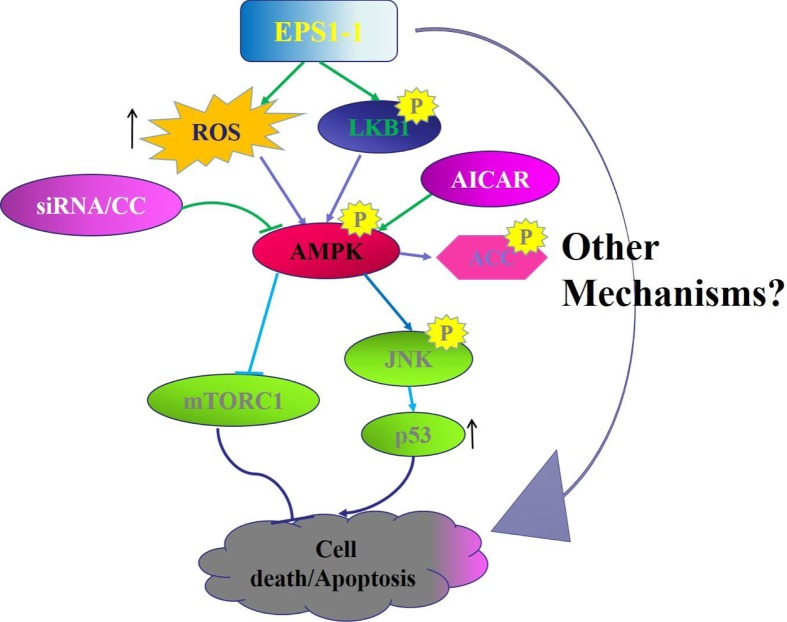
Proposed signaling pathway involved in EPS1-1 anti-cancer activity ROS and LKB1 may be required to activate AMPK by EPS1-1. EPS1-1 inhibits mTORC1 activation and activates the JNK-p53 signaling axis through AMPK activation and thus, EPS1-1 induces CT26 cell death/apoptosis.

The results of the present study indicated that EPS1-1 is a potential novel anti-cancer drug for CRC. *In vitro*, we determined that EPS1-1 contributes to reduced CT26 cell viability and activates the AMPK pathway. Moreover, we defined the two pathways responsible for the EPS1-1-enhanced apoptosis, namely the ROS, LKB1-AMPK-mTORC1 pathway and the ROS, LKB1-AMPK-JNK-P53 pathway. *In vivo*, we effectively induced CRC in BALB/c mice following treatment with AOM/DSS. However, this effect was significantly suppressed by EPS1-1 [25]. At the molecular level, our results suggest that EPS1-1 may activate the AMPK pathway, while inhibiting mTORC1 activity, and up-regulating the expression of p53 in tumor tissue. These results suggest that AMPK-regulated mTOR and JNK/p53 play a key role in EPS1-1-induced tumor cell apoptosis (Figure 10).

The purpose of our study was to confirm whether viability and apoptosis of CT26 cells were directly mediated by EPS1-1, and, which molecular mechanisms were responsible for any observed effects. The pivotal observation from the present study was that the AMPK pathway participates in EPS1-1-regulated reduced CT26 cell viability. AMPK is a major energy regulator in a wide range of species, from yeast to mammals, that functions to control glucose and lipid metabolism in response to changes in nutrients and intracellular energy levels. Moreover, activation of AMPK can shut down energy expending processes thereby inhibiting the pathways required for energy utilization in tumor cells [39–41]. Hence, a loss of AMPK signaling has been described in various cancer cells. AMPK activation may serve to alleviate disordered cellular metabolism in cancers cells and suppress tumorigenesis by inhibiting cellular proliferation and simultaneously inducing apoptosis in tumor cells. AMPK has, therefore, been proposed as a therapeutic approach to decrease tumorigenesis [17,42,43].

ROS can serve as a signaling molecular to activate AMPK, which inhibits cell growth and tumorigenesis [27]. We further explored the mechanism by which the production of ROS contributes to AMPK activation. A number of studies suggested that AMPK can be mediated by ROS [44,45] or by an increase in cellular AMP via allosteric mechanisms [46]. Moreover, mitochondrial functional status and ROS generation can alter AMPK activity by two parallel but independent pathways: redox signaling from mitochondria and changes in adenine nucleotides [47]. EPS1-1 induced accumulation of ROS, whereas NAC, an antioxidant, prevented the EPS1-1-induced decline in cell viability and inhibited the activation of AMPK, indicating that the anti-colon cancer effect of EPS1-1 relies on ROS induced AMPK activation. LKB1 is a tumor suppressor kinase and a major mediator of the cellular response to energy stress [48]. The activation of LKB1 is involved in various cellular functions, including cell proliferation, apoptosis and metabolism [49]. EPS1-1 may also activate AMPK signaling and reduce CT26 cell viability via an LKB1-dependent manner.

The mTOR pathway is also a key regulator of cell growth and proliferation, and in various cancers mTOR hyperactivation is often detected, which markedly contributes to cancer progression [32]. Therefore, mTOR signaling has been described as an important target for cancer diagnosis. Multiple reports have demonstrated that the activation of AMPK signaling is capable of suppressing mTORC1 activation [9,50,51]. This AMPK inhibitory effect on mTOR in tumor cells occurs by two distinct pathways: (1) AMPK phosphorylates TSC2 at Ser^1345^, which stimulates its Rheb-GAP activity; (2) AMPK phosphorylates Raptor at Ser^722^ and Ser^792^ which are both reasonably well conserved throughout eukaryotes [52–54]. TSC tumor suppressors are important upstream inhibitors of the mTORC1 complex [32,54] and as such are the most thoroughly and universally studied factor. Notably, the Raptor branch of the mTOR pathway modulates a stunning number of major cellular processes, including mRNA translation, ribosome biogenesis, nutrient metabolism, and autophagy [32]. Gwinn et al. [53] reported that the direct phosphorylation of Raptor by AMPK is sufficient for the inhibition of mTORC1 [52]. In this study, our results show that EPS1-1 induced the phosphorylation of AMPKα, leading to mTORC1 inhibition in CT26 cells. However, which mTOR associated pathway is involved in this inhibitory effect requires further exploration.

Additionally, AMPK activation induces the activation of JNK and pro-apoptotic p53, thereby inhibiting the growth of different tumors [55–57]. Moreover, the activation of JNK has been acknowledged as being necessary for the induction of apoptosis in different tumor cell types [58,59]. JNK may phosphorylate and regulate the activity of transcription factors such as p53 [60,61]. Specifically, c-Jun (the main target of JNK) is a direct repressor of p53 gene transcription [62]. A study has reported that in response to UV, c-Jun suppresses p53-mediated cell-cycle arrest to promote p53-regulated apoptosis [63]. In addition, CAA45 has been reported to exhibit potential anti-lung cancer effects via inhibition of Topo-I, resulting in cell-cycle arrest and reducing cell migration. Further, induction of mitochondria serves to regulate cellular apoptosis and autophagy via the PI3K/Akt/JNK/p53 pathway [56]. ASK1 is activated in response to various cytotoxic stresses, and activates JNK to induce tumor cell apoptosis [64]. Recent studies have reported that in cultured colon cancer cells, plumbagin induces AMPK/ASK1/TRAF2 association to activate the pro-apoptotic JNK-p53 signaling axis [65]. In the current study, we found that EPS1-1 activates the JNK-p53 signaling axis through activation of the AMPK pathway. However, we do not yet know whether there is a direct association between AMPK activation and EPS1-1. Furthermore, we recognize that AMPK may be one of many kinases that regulate the JNK/p53 activation in response to EPS1-1.

In summary, our results reveal that EPS1-1 triggers AMPK-mediated apoptosis by modulating mTOR signaling and the JNK/p53 signaling axis *in vitro* and *in vivo*. These findings indicate that EPS1-1 may be a potential anti-CRC drug through activation of the AMPK pathway.

## Supplementary Material

Supplementary Table S1

## Abbreviations

ACC: acetyl-coA carboxylase
AICAR: 5-Aminoimidazole-4-carboxamide-1-β-d-ribofuranoside
AMPK: AMP-activated protein kinase
AOM/DSS: azoxymethane/dextran sulfate sodium
ASK1: Apoptosis signal-regulating kinase 1
BALB/c: the variety of the mouse
CRC: colorectal cancer
DCF-DA: 6-Carboxy-2′,7′-dichlorofluorescein diacetate
DCFH-DA: 2′,7′-dichlorofluorescein diacetate
LKB1: liver kinase B1
mTORC1: mammalian target of rapamycin complex 1
MTT: 3-(4,5-dimethylthiazol-2-yl)-2,5-diphenyltetrazolium bromide
NAC: N-acetyl-l-cysteine
PBS: phosphate buffered saline
p70s6k: Ribosomal protein s6 kinase, 70 kda
Rheb-GAP: GTPase active protein, GAP
ROS: reactive oxygen species
RPMI: Roswell Park Memorial Institute
SEC: (S)-ethyl 1-(3-(4-chlorophenoxy)-2-hydroxypropyl)-3-(4-methoxyphenyl)-1 H-pyrazole-5-carboxylate
siRNA: small interfering RNA
SPF: Specefic Pathogen Free
TSC: Tuverous sclerosis complex
4E-BP1: e IF4E-binding protein 1
