# Supplementary material for: Exopolysaccharides isolated from Rhizopus nigricans induced colon cancer cell apoptosis in vitro and in vivo via activating the AMPK pathway
Source: Biosci Rep. 2020 Jan 14;40(1):BSR20192774. doi: 10.1042/BSR20192774 (PMC6960068; doi:10.1042/BSR20192774)
Supplement: Supplementary Table S1 [file BSR-2019-2774_supp.pdf]

Table S1 Primary antibodies.

**Antibodies**	**Types**	**Manufacturers**
AMPKα antibody	2531	Cell Signaling Technology
p-AMPKα antibody	2531	Cell Signaling Technology
LKB1 antibody	3047	Cell Signaling Technology
p-LKB1 antibody	3482	Cell Signaling Technology
ACC antibody	3662	Cell Signaling Technology
p-ACC antibody	3661	Cell Signaling Technology
β-actin antibody	4970	Cell Signaling Technology
mTOR antibody	2972	Cell Signaling Technology
p-mTOR antibody	2971	Cell Signaling Technology
4E-BP1 antibody	Ab32024	Abcam
p-4E-BP1 antibody	Ab75767	Abcam
p70s6k antibody	Ab9366	Abcam
p-p70s6k antibody	Ab60948	Abcam
JNK antibody	9252	Cell Signaling Technology
p-JNK antibody	9251	Cell Signaling Technology
p53 antibody	2524	Cell Signaling Technology
Caspase-3 antibody	9662	Cell Signaling Technology
